# Supplementary material for: Ligamentization of the reconstructed ACL differs between the intraarticular and intraosseous regions: A quantitative assessment using UTE-T2* mapping
Source: PLoS One. 2022 Jul 22;17(7):e0271935. doi: 10.1371/journal.pone.0271935 (PMC9307199; doi:10.1371/journal.pone.0271935)
Supplement: Attachment — Submitted filename: Response to Reviewers.docx [file pone.0271935.s002.docx]

PONE-D-22-05680

Ligamentization of the reconstructed ACL differs between the intraarticular and intraosseous regions: A quantitative assessment using UTE-T2* mapping

PLOS ONE

Dear Dr. Nakase,

Thank you for submitting your manuscript to PLOS ONE. After careful consideration, we feel that it has merit but does not fully meet PLOS ONE’s publication criteria as it currently stands. Therefore, we invite you to submit a revised version of the manuscript that addresses the points raised during the review process.

Please submit your revised manuscript by Jun 09 2022 11:59PM. If you will need more time than this to complete your revisions, please reply to this message or contact the journal office at [plosone@plos.org](mailto:plosone@plos.org). When you're ready to submit your revision, log on to https://www.editorialmanager.com/pone/ and select the 'Submissions Needing Revision' folder to locate your manuscript file.

Please include the following items when submitting your revised manuscript:

- A rebuttal letter that responds to each point raised by the academic editor and reviewer(s). You should upload this letter as a separate file labeled 'Response to Reviewers'.
- A marked-up copy of your manuscript that highlights changes made to the original version. You should upload this as a separate file labeled 'Revised Manuscript with Track Changes'.
- An unmarked version of your revised paper without tracked changes. You should upload this as a separate file labeled 'Manuscript'.

If you would like to make changes to your financial disclosure, please include your updated statement in your cover letter. Guidelines for resubmitting your figure files are available below the reviewer comments at the end of this letter.

If applicable, we recommend that you deposit your laboratory protocols in protocols.io to enhance the reproducibility of your results. Protocols.io assigns your protocol its own identifier (DOI) so that it can be cited independently in the future. For instructions see: <https://journals.plos.org/plosone/s/submission-guidelines#loc-laboratory-protocols>. Additionally, PLOS ONE offers an option for publishing peer-reviewed Lab Protocol articles, which describe protocols hosted on protocols.io. Read more information on sharing protocols at <https://plos.org/protocols?utm_medium=editorial-email&utm_source=authorletters&utm_campaign=protocols>.

We look forward to receiving your revised manuscript.

Kind regards,

Markus Geßlein, Ph.D.
Section Editor
PLOS ONE

Journal Requirements:

1. When submitting your revision, we need you to address these additional requirements.

Please ensure that your manuscript meets PLOS ONE's style requirements, including those for file naming. The PLOS ONE style templates can be found at 
https://journals.plos.org/plosone/s/file?id=wjVg/PLOSOne_formatting_sample_main_body.pdf and 
https://journals.plos.org/plosone/s/file?id=ba62/PLOSOne_formatting_sample_title_authors_affiliations.pdf

2. Please include captions for your Supporting Information files at the end of your manuscript, and update any in-text citations to match accordingly. Please see our Supporting Information guidelines for more information: http://journals.plos.org/plosone/s/supporting-information.

Additional Editor Comments:

Dear Authors,

thank you very much for submitting your well-performed study to PlosOne. After review by two experienced colleagues in this field I would kindly ask your for some improvments in your manuscript. You will find the detailled reviewers comments below.


Furthermore, I would kindly ask you to adress the additional issues below. I think this will make your manuscript more interesting for a broader readership.

**Response:** Thank you for your review and valuable comments and suggestions. We have attempted to address every concern in the revised manuscript. Our point-by-point responses to each comment are given below.

Abstract

Conclusion

- Is a bit of repetition according to the abstracts result section? Please provide any potential clinical benefit of your study or describe why this finding could be important for future research (see comment on discussion below).
- Conclusion in the abstract and the manuscript should be same.
**Response:** Thank you for pointing this out. We have revised the conclusions to highlight the potential clinical benefits of the study in the Abstract (Page 3, Lines 39) as well as main text (Page 21, Lines 377). We have also ensured consistency between the conclusions in the Abstract and those in the main text.

Material & Methods

-Please provide more information about the female patients included e.g. BMI, Height, side of injury, time from injury to surgery, maybe injury mechanism etc..
**Response:** Thank you for the recommendations. We have added the required information in the Patients and Methods section (Page 8, Lines 119).

Discussion

-The first paragraph of the discussion should contain the most important findings of your study. I would suggest using the actual conclusion as first paragraph for the discussion. Then rewriting the conclusion with reference to the clinical importance or future research topics you would suggest.
**Response:** Thank you for pointing this out. We have now revised the first paragraph of the Discussion section (Page 15, Lines 226) and the Conclusions section (Page 21, Lines 343) in accordance with your suggestions.

-I would like to make the manuscript more interesting to read for orthopedic surgeons having to deal with the RTA/RTS issues in daily routine.

-I would like to ask you to move figure 3 to the results section. Further, please explain in the methods section how “normal ACL” values were aquired.
**Response:** Thank you for your suggestions. We have moved Figure 3 to the Results section (Page 12, Lines 184) and added a reference citation pertaining to the measurement of normal ACL values in the Methods section (Page 10, Lines 148).

Limitations

-You did not provide data about the stability of knee after surgery. Orthopedic Surgeons well know that technical errors during surgery may result in decreased stability and that might affect the biological graft transformation. If you could provide data about the knee stability (clinical like Lachmann or KT 1000) that would improve the findings. If you don not have that data please mention this fact in the limitations.

**Response:** Thank you for the valuable insights. We are currently investigating the association of UTE-T2* values with clinical and functional outcomes (IKDC score and KOOS at 1 year postoperatively) of patients, including the subjects of the present study. Data regarding knee stability after surgery were insufficient in the present study. In the limitations section of the current manuscript, we have mentioned that investigation of the relationship of UTE-T2* values with clinical and functional outcomes is also important to provide ideas on safe return to sports in terms of maturation of reconstructed ACLs (Page 20, Lines 327).

-Please add information how the choice of enrolled participants could have influenced the results (only females..time between injury and surgery etc..)
**Response:** Thank you for pointing this out. We agree that selection bias is a concern in this study. We have mentioned this in the limitations paragraph and highlighted that future studies involving larger sample sizes and incorporating covariates that may affect the maturity of reconstructed ACLs are warranted (Page 20, Lines 328).

-Did metal remnants from tunnel drilling interfere with the scan /measures?
**Response:** Thank you for the pertinent question. If metal remnants were present, they would have a significant impact on the measurement as large artifacts. There were no such cases in the present study.

[Note: HTML markup is below. Please do not edit.]

Reviewers' comments:

Reviewer's Responses to Questions

**Comments to the Author**

1. Is the manuscript technically sound, and do the data support the conclusions?

The manuscript must describe a technically sound piece of scientific research with data that supports the conclusions. Experiments must have been conducted rigorously, with appropriate controls, replication, and sample sizes. The conclusions must be drawn appropriately based on the data presented.

Reviewer #1: Yes

Reviewer #2: Yes

2. Has the statistical analysis been performed appropriately and rigorously?

Reviewer #1: Yes

Reviewer #2: Yes

3. Have the authors made all data underlying the findings in their manuscript fully available?

The [PLOS Data policy](http://www.plosone.org/static/policies.action#sharing) requires authors to make all data underlying the findings described in their manuscript fully available without restriction, with rare exception (please refer to the Data Availability Statement in the manuscript PDF file). The data should be provided as part of the manuscript or its supporting information, or deposited to a public repository. For example, in addition to summary statistics, the data points behind means, medians and variance measures should be available. If there are restrictions on publicly sharing data—e.g. participant privacy or use of data from a third party—those must be specified.

Reviewer #1: Yes

Reviewer #2: Yes

4. Is the manuscript presented in an intelligible fashion and written in standard English?

PLOS ONE does not copyedit accepted manuscripts, so the language in submitted articles must be clear, correct, and unambiguous. Any typographical or grammatical errors should be corrected at revision, so please note any specific errors here.

Reviewer #1: Yes

Reviewer #2: Yes

5. Review Comments to the Author

Please use the space provided to explain your answers to the questions above. You may also include additional comments for the author, including concerns about dual publication, research ethics, or publication ethics. (Please upload your review as an attachment if it exceeds 20,000 characters)

Reviewer #1: In this manuscript, authors investigated the maturation of reconstructed ACL using UTE-T2* imaging. This is a well-written manuscript. I do have some minor concerns:

1. Statistical Analysis: Only pairwise comparison of T2* values was conducted at three time points. As demonstrated in Figure 2, there are some clear trends of T2* over time, especially in intraarticular region. Correlation T2* values with recovery time would be interesting.
2. In Discussion, authors claimed that this is the first study to investigate regional graft maturation with UTE. There is at least one recent publication in this topic: Fukuda T, et al, Abbreviated quantitative UTE imaging in anterior cruciate ligament reconstruction, BMC Musculoskeletal Disords. 2019;20(1):426;

**Response:** Thank you for pointing that out. As you pointed out, previous studies have measured the T2* values of reconstructed ACLs separately in the joint and in the bony foramen. We have deleted the word “first” and rephrased the sentence in the revised manuscript (Page 15, Lines 226 and Page21, Lines 337).

3. In Discussion, authors claimed the long acquisition duration needed for bi-exponential UTE study. It would be true if using fully-sampled acquisition. As described in Fukuda et al paper, it is possible to reduce the time by partial acquisition.
4. The confounds of mono-exponential instead of bi-exponential should be discussed. The fraction of bound water is quite high in both reconstructed and healthy ACLs. The inclusion of first echo (TE=0.1 ms) may led to errors in mono-exponential T2* estimation. A comparison of results between using all 4 TEs vs. using only 3 TEs (excluding first TE) would be meaningful.

**Response:** Thank you for your valuable comments and suggestions. We have mentioned in the limitations that biexponential UTE-T2* mapping was deemed inappropriate for routine clinical MRI in this study because of its long acquisition time, although partial acquisition could have reduced the time. We have also discussed the confounds of using mono exponential mapping instead of biexponential mapping in the limitations (Page 20, Lines 321).

5. As indicated in literature, there are significant changes during the first 6 months post-op. Some discussion/clarification are needed.

**Response:** Thank you for the helpful comments. We have discussed the main histological findings pertaining to the changes during the first 6 months after surgery in the Discussion section (Page 16, Lines 249).

Reviewer #2: In the present prospective study, the authors Rikuto Y et al. use a UTE T2* mapping technique on a defined patient collective (10 female patients, 18 +/- 4 years of age) to assess the UTE T2* value changes within the tendon graft in the first postoperative year after ACL reconstruction with regards to the histological process of ligamentisation. The progressing ligamentisation has been shown to correlate with an increasing stability of the ACL reconstruction (although mainly in animal trials), which is an important factor with regards to return to physical activity as well as to avoiding the risk of early graft re-tear.

In correlation with other recent studies by Chu CR et al (Orthop J Sports Med. 2019) and Warth RJ and al. (Am J Sports Med. 2020), the presented UTE T2* values of the intraarticular segment of the ACL grafts drop significantly between 6 to 9 months postoperatively, without another significant change in the last quarter of the first postoperative year. The study also assesses the UTE T2* values of the femoral and tibial intraosseous segments of the ACL grafts, which are significantly lower than the intraarticular UTE T2* values, especially 6 months postoperatively. Compared to previously assessed UTE T2* values in uninjured ACL (see Okuda M et al., Acta Radiol. 2021), the intraosseous graft segments present similar values. In addition, a significant difference of UTE T2* values between the femoral and the tibial intraosseous segments of the ACL grafts is noted.

The authors conclude that the ligamentisation processes in the intraarticular and intraosseous segments of the ACL reconstructions differ significantly within the first 6 months postoperatively with a slowly increasing convergence after 9 to 12 months, which points towards a significant tendency for faster maturation intraosseously. Moreover, a significant difference is described in ligamentisation of the intraosseous segments in the first postoperative year, which is discussed to possibly be in relation to tunnel motion.

The authors present their findings and conclusions, as well as the study’s limitations, in a clear and concise manner. The correlating histological and imaging findings by other studies, added in the introduction and the discussion, give weight to the study’s overall conclusion.

The patient collective is explained to have been selected prospectively and includes patients operated on by one orthopaedic surgeon with a precisely explained surgical method. While the patient collective is small, consisting of just one gender and limited to a certain age, it is within the limit of the predetermined, calculated sample size.

The technique used for imaging acquisition, including its limitations (e.g. metal artefacts, magic angle effect), is explained in detail and is easily understandable, both in the introduction and in the method section. The measurement method as well as the statistical evaluation are well described and not too technical. As a radiologist, I especially appreciate the precise explanations with regards to the differences in MRI imaging and MRI imaging acquisition concerning this kind of tissue material.


In the last few years, mapping techniques in MR imaging have gained importance in the diagnostic assessment of tissue characteristics, and in particular of tissue pathologies (e.g. T1/T2 mapping of myocardium in cardiac imaging). UTE sequences have been increasingly applied in the diagnostics of the musculoskeletal system, given that they can reveal alterations in tissues with short to ultra-short transverse relaxation times, such as bone, ligaments or tendons. Tissues with moderate to long transverse relaxation times, e.g. cartilage, can already be reliably assessed with conventional MRI sequences (see also Chang E. et al, Journal of Magnetic Resonance Imaging 41:870–883 (2015)).

From a radiologist’s point of view, studies like the present authored by Rikuto Y et al. may give rise to more detailed studies, which will hopefully contribute to establishing these type of measuring methods in the daily routine of MRI imaging. The aforementioned is subject to the condition that the correlation between measured values and biomechanical changes prove to be significant to the clinical outcome of patients.
**Response:** Thank you for your detailed review and positive feedback for our study. We are currently investigating the association of UTE-T2* values with clinical and functional outcomes (IKDC score and KOOS at 1 year postoperatively) of patients, including the subjects of the present study. We will analyze and present the results once a sufficient sample is collected.

No major issues can be observed in the manuscript.

However, following minor issues could be considered:

- Page 12, Line 193: The measurements were taken by one orthopaedic surgeon as defined in the methodology. I found no mention of other evaluators in the text. It is not completely clear to me how the interobserver reliability could be assessed precisely in the results.

**Response:** We apologize for the lack of clarity regarding interobserver reliability. We have now mentioned in the Patients and Methods section that another orthopedic surgeon (YY, Observer 2) independently performed measurements using the same method in order to assess interobserver reliability (Page 10, Lines 155).

- Page 10 Line 156-160, Page 13/14 Line 207-211, Page 18 Line 281-284: The same, detailed passages of measurement and result explanations, which are included already in the adjacent text, can be found underneath the figures’ titles. They read repetitive, especially since the figures aren’t shown within the text in the downloaded PDF version. In my opinion, captions of figures should be kept succinct.

**Response:** Thank you for pointing this out. We have revised each caption to be more concise.

- Page 16 Line 248-252 / Page 17 Line 267-270: Another exact text passage repetition. While both passages fit within the context of the discussion, their proximity within the text impacts the fluency of the narrative. Paraphrasing of one passage may be better.

**Response:** Thank you for pointing this out. We have now deleted and rearranged some text in the repetitive paragraph (Page 17-18, Lines 266-289).

6. PLOS authors have the option to publish the peer review history of their article ([what does this mean?](https://journals.plos.org/plosone/s/editorial-and-peer-review-process#loc-peer-review-history)). If published, this will include your full peer review and any attached files.


If you choose “no”, your identity will remain anonymous but your review may still be made public.


**Do you want your identity to be public for this peer review?** For information about this choice, including consent withdrawal, please see our [Privacy Policy](https://www.plos.org/privacy-policy).

Reviewer #1: **Yes:** Xiang He

Reviewer #2: **Yes:** Dr. Maria Elena Misu

[NOTE: If reviewer comments were submitted as an attachment file, they will be attached to this email and accessible via the submission site. Please log into your account, locate the manuscript record, and check for the action link "View Attachments". If this link does not appear, there are no attachment files.]

While revising your submission, please upload your figure files to the Preflight Analysis and Conversion Engine (PACE) digital diagnostic tool, <https://pacev2.apexcovantage.com/>. PACE helps ensure that figures meet PLOS requirements. To use PACE, you must first register as a user. Registration is free. Then, login and navigate to the UPLOAD tab, where you will find detailed instructions on how to use the tool. If you encounter any issues or have any questions when using PACE, please email PLOS at [figures@plos.org](mailto:figures@plos.org). Please note that Supporting Information files do not need this step.
